# Ethiopia National Cholera Elimination Plan 2022–2028: Experiences, Challenges, and the Way Forward

**DOI:** 10.1093/cid/ciae200

**Published:** 2024-07-12

**Authors:** Mukemil Hussen, Yeshambel Worku Demlie, Moti Edosa, Mandefro Kebede, Mesfin Wossen, Azeb Mulugeta Chane, Girma Abate, Wondosen Hailu Asfaw, Dejene Hailu, Mekonnen Teferi, Yeonji Jeon, Abel Gedefaw, Se Eun Park

**Affiliations:** Public Health Emergency Management, Ethiopia Public Health Institute, Addis Ababa, Ethiopia; Public Health Emergency Management, Ethiopia Public Health Institute, Addis Ababa, Ethiopia; Public Health Emergency Management, Ethiopia Public Health Institute, Addis Ababa, Ethiopia; Public Health Emergency Management, Ethiopia Public Health Institute, Addis Ababa, Ethiopia; Public Health Emergency Management, Ethiopia Public Health Institute, Addis Ababa, Ethiopia; Public Health Emergency Management, Ethiopia Public Health Institute, Addis Ababa, Ethiopia; Public Health Emergency Management, Ethiopia Public Health Institute, Addis Ababa, Ethiopia; Public Health Emergency Management, Ethiopia Public Health Institute, Addis Ababa, Ethiopia; Clinical, Assessment, Regulatory, and Evaluation Unit, International Vaccine Institute, Seoul, Republic of Korea; School of Public Health, Hawassa University, Hawassa, Ethiopia; Clinical Trials Directorate, Armauer Hansen Research Institute, Addis Ababa, Ethiopia; Clinical, Assessment, Regulatory, and Evaluation Unit, International Vaccine Institute, Seoul, Republic of Korea; Clinical, Assessment, Regulatory, and Evaluation Unit, International Vaccine Institute, Seoul, Republic of Korea; College of Medicine and Health Sciences, Hawassa University, Hawassa, Ethiopia; Clinical, Assessment, Regulatory, and Evaluation Unit, International Vaccine Institute, Seoul, Republic of Korea; Department of Global Health and Disease Control, Yonsei University Graduate School of Public Health, Seoul, Republic of Korea

**Keywords:** Cholera, Ethiopia, National Cholera Elimination Plan, NCP, Cholera Plan

## Abstract

Cholera remains a significant public health concern in Ethiopia. More than 15.9 million Ethiopians, constituting 15% of the total population, live in areas with a history of recurrent cholera outbreaks. The last 9 years of national cholera surveillance data show the country has been experiencing cholera outbreaks every year. The current cholera outbreak, starting in August 2022, has affected the entire country, with 841 reported cases and a 3.13% case fatality rate (CFR) in 2022, and >30 000 cases with nearly a 1.4% CFR in 2023. In line with “Ending Cholera—A Global Roadmap to 2030,” the government of Ethiopia is committed to eliminate cholera in the country and has prepared its “National Cholera Elimination Plan (NCP): 2022–2028” with aims to achieve zero local transmission in cholera hotspot areas by 2028 and 90% fatality reduction from the recent (2020–2022) average of 1.8% CFR. The plan is multisectoral, has a clear coordination platform, contains all interventions with in-depth situational analysis, is concordant with existing plans and strategies, and is cascaded at the regional level and implemented with existing government and public structures. Nationwide, total 118 cholera hotspot woredas (districts) were identified, and a comprehensive situation analysis of the existing cholera outbreak response capacity was assessed. This multisectoral and multiyear NCP has forecasted around US$404 million budget estimates with >90% allocated to improving the country's water, sanitation, and hygiene (US$222 million; 55% of total NCP budget) and case management (US$149 million; 37%). The cholera vaccination strategy included in the NCP exhibited a 5-year oral cholera vaccine (OCV) introduction plan with 2 doses (30 604 889 doses) and single dose (3 031 266 doses) in selected cholera hotspot areas. However, its implementation is challenged due to a lack of financial support, inability to get the requested vaccine for targeted hotspot woredas (due to the current shortage of doses in the OCV global stockpile), recurrent cholera outbreaks, and high humanitarian needs in the country. It is recommended to have a sustainable financial mechanism to support implementation, follow the requested vaccine doses, and reorganize the planned coordination platform to foster the implementation.

Cholera remains a significant global public health concern. Approximately 1.3 billion people worldwide are at risk, resulting in an annual incidence of 2.9 million cases and 95 000 deaths [1]. Ethiopia, a country with a long history of endemic and epidemic cholera, has faced persistent challenges. Although well-organized cholera data at the national level in Ethiopia became available after 2015, available evidence in the past 2 decades (2001–2023) reported a total of 215 205 acute watery diarrhea/cholera cases and 2355 deaths in the country [2]. In the years 2006, 2007, 2009, 2016, 2017, and 2023, there was a notable surge in case counts and attack rates, with each year exceeding 20 000 acute watery diarrhea/cholera cases annually. The peak cholera outbreak years coincide with the Eastern Africa El Niño events in 2006–2007, 2015–2016, and 2023, indicating a potential impact of climate change on cholera epidemics [3].

Over the recent 9 years (2015–2023), Ethiopia has consistently faced cholera outbreaks, reporting a total of 99 945 cases and 1030 deaths according to the Ethiopia Public Health Institute (EPHI) national cholera line-list [2]. The first epidemic of this period started in late 2015 (in the Oromia and Somali regions) and spread throughout the country in 2016. Cholera outbreaks in Ethiopia reached their peak in 2016 (29 871 cases) and 2017 (20 509 cases), followed by a decrease in the subsequent 2 years. However, even during these relatively lower periods, annual cases remained in the thousands: 3259 cases in 2018 and 2271 cases in 2019. The year 2020 witnessed a significant resurgence with 15 167 cases, while 2021 and 2022 saw a decline in reported cases during the coronavirus disease 2019 (COVID-19) pandemic (696 and 841 cases, respectively). A notable peak reemerged in the 2023 cholera outbreak with nearly 30 000 cases, marking the highest incidence in the past 5 years. Corresponding to the heightened outbreak in 2023 in Ethiopia, the World Health Organization (WHO) also observed a global resurgence of cholera after several years of decline [4].

Despite the observed multiyear temporal variation in the magnitude of cholera outbreaks in Ethiopia, the most recent national cholera outbreak data (2021–2023) revealed an elevated cholera case fatality rate (CFR), averaging 1.87% [2]. The recent cholera outbreak that commenced in August 2022 recorded the highest CFR, reaching 3.13% (with a 95% confidence interval of 2.1%–4.5%). During the ongoing cholera outbreak in 2023 and amid the 2020 COVID-19 pandemic, a CFR of approximately 1.4% was documented [2], surpassing the WHO-recommended threshold of 1% [5–7].

In response to the cholera outbreak in August 2022, the Ethiopian government, with partners, set up around 307 cholera treatment centers/units (CTCs) in the affected woredas (districts). Healthcare workers received training to enhance case management and reduce fatalities, focusing on individual patient dehydration levels according to the national guideline. Patients with moderate to severe dehydration are admitted to CTCs for intravenous fluids, while those with mild dehydration are managed with oral rehydration therapy at home or in a CTC, depending on the CTC caseloads. The National Cholera Elimination Plan (NCP) aims to decrease the CFR by addressing the identified infrastructure and resource gaps that resulted in high mortality, especially in remote areas. In addition to the water, sanitation, and hygiene (WaSH) and risk communication and community engagement (RCCE) interventions, the government has implemented several reactive oral cholera vaccine (OCV) mass vaccination campaigns during the recent series of cholera outbreaks. From 2019 until November 2023, the International Coordinating Group approved >20 million doses of OCV from the global stockpile, of which around 18 million doses were delivered in Ethiopia and >16 million doses administered [8].

## GOVERNMENT COMMITMENT TO CHOLERA OUTBREAK CONTROL AND PREVENTION

In response to these cholera outbreaks, the government has undertaken a series of measures for cholera control and prevention. To harmonize the outbreak responses at the regional level, the initial Guideline on Cholera Outbreak Control and Management, developed in 2011 [9], has undergone revisions in 2022 [10]. The initial guideline focused on the national cholera surveillance system, emphasizing case detection, outbreak investigation, and the management and reporting of cases [9]. The revised guideline aimed to enhance cholera control and management, specifically addressing the recent (2019–2022) challenges faced by internally displaced populations, emphasizing the importance of cross-border collaboration for effective cholera control and prevention, and lowering the age threshold for cholera case definition criteria in outbreaks (from 5 years to 2 years) [10]. The revised guideline further incorporates OCV as a crucial prevention strategy.

The integration of OCV is also elaborated in the WHO regional framework for the implementation of the global strategy for cholera prevention and control [11] and the Ethiopian government's NCP [12]. The cholera vaccination strategy included in the NCP exhibited a 5-year OCV plan with 2-dose (30 604 889 doses) and single-dose (3 031 266 doses) vaccination in selected cholera hotspot areas [12]. However, most OCV doses have been used in response to cholera outbreaks in a series of reactive mass vaccination campaigns using a single-dose strategy. A total of 15 OCV mass vaccination campaigns were conducted during 2019 and 2023, comprising 12 reactive and 3 preemptive campaigns [8, 13]. Overall, high administrative coverage of these vaccination campaigns was shown, but proper assessment of OCV coverage is needed to better document and understand the proportion and level of populations protected against cholera. Furthermore, it is important to note that the 2021 OCV vaccination campaign in the Tigray conflict region reported only 41.8% administrative coverage in the first round (and no second-round vaccination was carried out), highlighting the challenges faced in areas affected by conflict [8].

## ETHIOPIAN NCP DEVELOPMENT EXPERIENCES AND LESSONS LEARNED

Ethiopia, along with the other WHO member states, passed a resolution (WHA71.4) at the 71st World Health Assembly in 2018. The resolution urges the affected state parties to take cholera as a priority disease, integrate it into their national plans, use a multisectoral approach, and endorse the global initiative to end cholera [14]. The Global Roadmap to End Cholera aims to reduce cholera-related deaths by 90% and eliminate cholera in at least 20 countries by 2030 [15]. On 23 July 2019, the Ethiopian government expressed its commitment to the initiative during the multisectoral stakeholder meeting “High-Level Briefing on the Global Roadmap on Cholera Elimination 2030.” The State Minister of the Ministry of Health (MoH) and the Minister of Water, Irrigation and Electricity pledged to adopt the framework and engage on the principles of the roadmap by agreeing to take evidence-based actions, which included enhancing epidemiological and laboratory surveillance to map cholera hotspots, improving access to timely treatment, promoting community engagement, integrating the use of OCV, and increasing investment in safely managed water and sanitation and improving hygienic conditions or behaviors for the most vulnerable communities [16]. Following this high-level meeting, the government of Ethiopia, with the leadership of the EPHI, took the initiative in preparing the NCP 2022–2028. A multisectoral team was set up with core stakeholders from the MoH Expanded Program on Immunization, MoH Emergency, Water, Sanitation, and Hygiene (WaSH) Section, Ministry of Water and Energy, Ministry of Finance, Ministry of Planning and Development, Disaster Risk Management Commission (DRMC), and other relevant governmental sectors and partners [12]. The Ethiopian NCP preparation took 3 years (2019–2021) and included workshops and consultations with stakeholders, partners, and regional health bureau staff.

### Multisectoral Approach

While the health sector has usually taken the lead in cholera outbreak preparedness and response to control immediate outbreaks, this unilateral effort failed to address the underlying causes of recurrent outbreaks. The lack of comprehensive engagement with other sectors has contributed to the persistence of cholera outbreaks. Recognizing this critical gap and the necessity for a coordinated and multisectoral approach to control cholera, the NCP was formulated through collaborative efforts involving various line ministries in addition to the MoH, nongovernmental organizations, health and WaSH partners, and donors. The NCP planning process involved 11 stakeholders from the government side and >7 partners, including WaSH cluster. During the high-level advocacy and consultative meetings on NCP development, the Health Minister, Her Excellency Dr. Lia Tadesse, addressed the importance of addressing cholera in coordinated actions: “We are acutely aware that cholera is not only a health problem, but a disease rooted in socioeconomic conditions such as access to clean water, hygiene, and sanitation. Therefore, the MoH is collaborating with other sectors and partners to control, and eventually eliminate, cholera from Ethiopia” [17].

### High-level Coordination Platform

The plan has a clear proposed coordination platform. The EPHI has taken the initiative to facilitate the formation of a National Health Security Council (NHSC), which is to be chaired by the Deputy Prime Minister to oversee the implementation of the National Action Plan for Health Security (NAPHS) [12]. While the formation of the NHSC is under discussion, implementing the NCP in the interim is proposed to be managed through the Disaster Risk Management Council.

### Situational Analysis

The targets and strategies have been meticulously formulated through a thorough analysis of the strengths, weaknesses, opportunities, and threats associated with the existing coordination and leadership mechanisms in public health emergencies, as well as the success and challenges gained from past experiences in cholera surveillance and case management. The situation analysis further integrated lessons learned from the 2019–2022 OCV campaigns, along with valuable insights from prior RCCE practices. Additional considerations also included the current status and ongoing projects of WaSH. Drawing on this extensive analysis, the NCP targets and strategies were developed with an aim to strategically address key areas for improvement and enhance the overall effectiveness of cholera control efforts.

### Cholera Hotspots and Case Management

**Figure 1.**
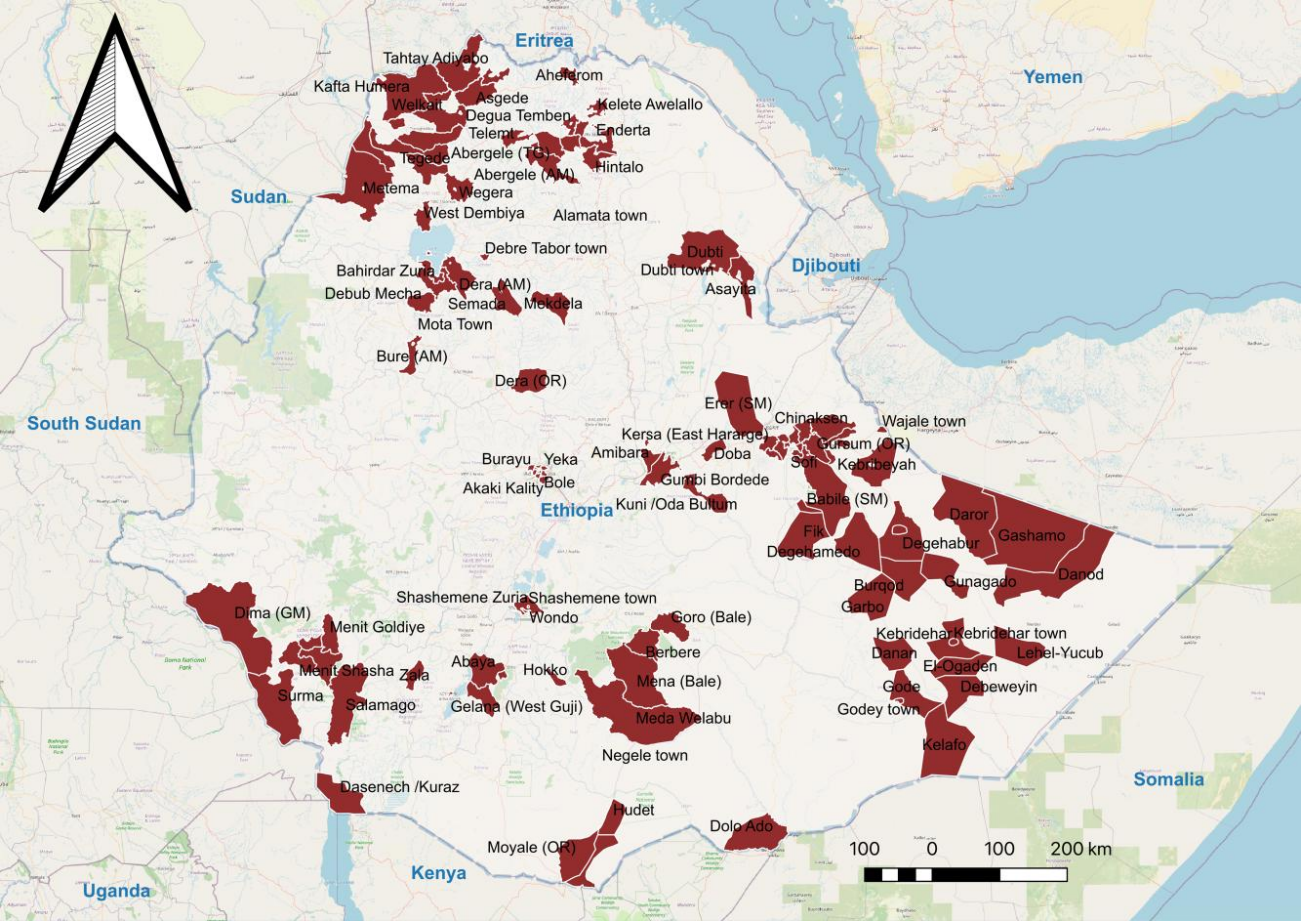
Map of identified cholera hotspots in Ethiopia.

The NCP includes 118 cholera hotspot woredas (Figure 1) with an estimated population of approximately 17.7 million based on the mean annual incidence and persistence of cholera cases in the past 5 years [12]. An annual incidence rate of >100 cases per 100 000 populations was considered high incidence, and the occurrence of cholera cases in 5% or higher of weeks was regarded as high persistence [18]. On case management, the primary support for cholera patients is to get proper medication. The NCP aims to prevent fatality through expanding treatment facilities and oral rehydration points in affected communities. Early case detection is a critical component of NCP. It involves identifying and diagnosing cholera cases as early as possible, including confirmation at a peripheral level.

### Concordance With Other Health Sector Plans

The NCP is strategically aligned with several key national frameworks, including the Growth and Transformation Plan II (GTP-II), Health Sector Transformation Plan II (HSTP-II) for 2021–2025, NAPHS, One WaSH Plan, and other pertinent national strategies and initiatives. Additionally, it is in accordance with Ethiopia’s global commitments, specifically the Sustainable Development Goals. The GTP-II encompasses 21 goals and 4 strategic objectives directly linked to WaSH, reinforcing the importance of WaSH in national development. The NAPHS, crafted based on the 2016 Joint External Evaluation findings, prioritizes cholera as the primary disease of concern. These overarching plans and strategies address various hazards for comprehensive system improvement. The NCP maintains a cholera-specific focus and is primarily executed in cholera hotspot woredas [19].

### NCP Cascading at the Regional Level and Integrating With Existing Health Structure System

Cholera hotspot woredas were identified nationally with inputs from subnational level health bureaus. The planning of the NCP rollout has been conducted since February 2023, which helped to contextualize the NCP at the regional level, strengthen the regional ownership of the plan, and share activities and resources to monitor and evaluate the implementation of the NCP in respective regions across the country. The NCP implementation upholds the existing health structure, harmonized with the government's health system for the implementation, which helps to capacitate the system for prevention, early detection and reporting, and response to cholera outbreaks and other public health emergencies.

### NCP Strategic Objectives and Targets

**Figure 2.**
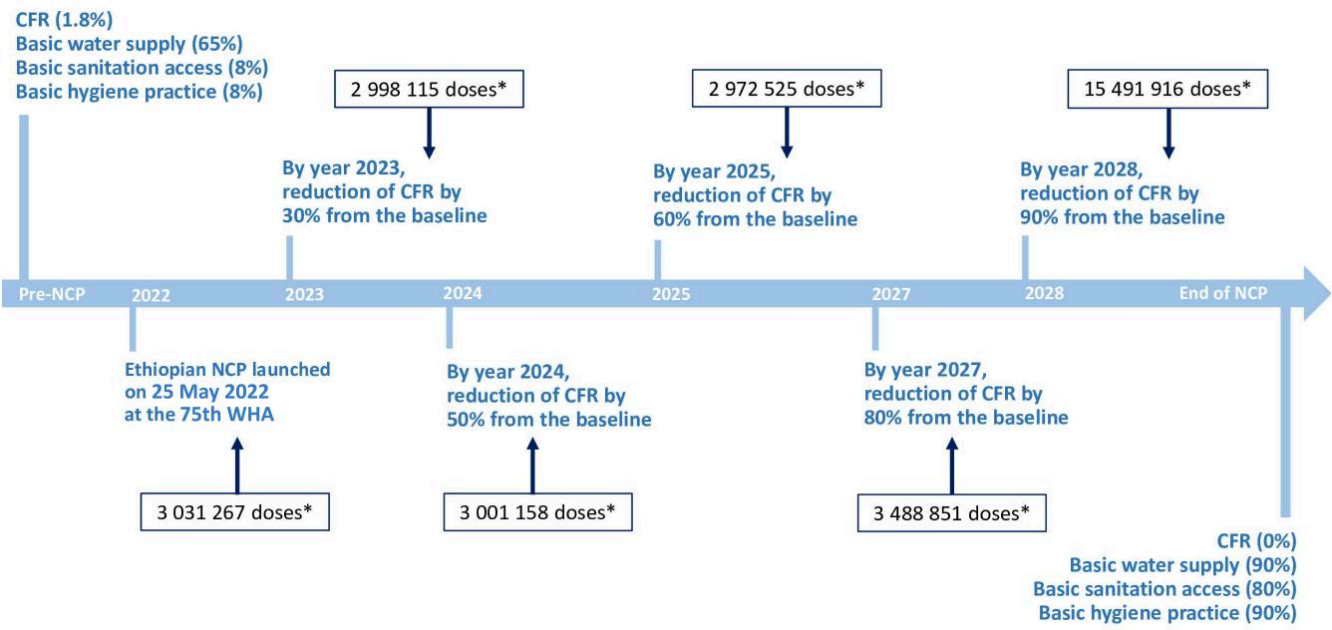
Ethiopia National Cholera Elimination Plan (NCP) target goals and timeline. *Doses for 2-dose preventive oral cholera vaccination campaign planned in the NCP. Abbreviations: CFR, case fatality rate; NCP, National Cholera Elimination Plan; WHA, World Health Assembly.

The plan has 18 strategic objectives and 6 main targets (Figure 2). The main targets by pillars are (1) to have an effective leadership and coordination for cholera elimination under the Office of the Deputy Prime Minister; (2) to improve surveillance and laboratory capacity at all levels for early detection and confirmation of cholera cases by 2028; (3) to reduce the overall mortality resulting from cholera by 90% by 2028 and ensure that there is no local transmission of cholera reported in the 118 hotspot districts; (4) to improve access to basic water supply, sanitation, and hygiene at all levels of high-risk kebeles (wards) within cholera hotspot woredas to eliminate cholera in hotspots (such as increasing basic water supply from 65% to 90% and improving sanitation and hygiene coverage from 6% to 80% by 2028); (5) to conduct OCV vaccination campaigns (2 doses within at least 2 weeks and no more than 6-month dose intervals) with vaccination coverage of >90% in cholera hotspots and outbreak areas; and (6) to mainstream community engagement into all pillars to assure sustainability of interventions for the elimination of cholera.

## CHALLENGES AND WAY FORWARD

### Lack of Comprehensive National Cholera Data for Identifying Hotspots

Obtaining at least 5 years of cholera data was a requirement to prioritize hotspot woredas. Finding data from 2015 and backward was challenging as data had not been adequately stored in the National Data Center. However, the data were collected and collated from the regional, zonal, and woreda health offices and the WHO Country Office.

### COVID-19 Pandemic Impact

Ethiopia reported its first cases of COVID-19 in March 2020. In response to the WHO’s declaration of COVID-19 as a public health emergency, the MoH/EPHI activated an emergency operating center for preparedness and response even before the first case was reported. The impact of the COVID-19 pandemic on the national cholera outbreak and control has not been thoroughly investigated. Despite a general decline in cholera cases during 2021 and 2022 (<1000 cases in each year), recorded cholera outbreaks persisted throughout 2020–2022, with a peak of 15 167 cases in 2020 [2]. The decreased number of cholera cases could be attributed to COVID-19 interventions such as social isolation and the promotion of good hygiene practices. The geographical distribution of the outbreaks was localized in the southern and eastern parts (Oromia, Somali, and Southern Nations, Nationalities, and People’s regions), sparing the northern part of Ethiopia, without a clear justification for the association with the COVID-19 pandemic.

However, during the COVID-19 epidemic, the highest cholera CFRs were reported in 2022 (3.13%) and 2020 (1.4%) [2]. Delayed health-seeking behavior of cholera patients due to perceived COVID-19 infection in the healthcare facilities and prioritization of limited resources to COVID-19 response may have resulted in a poor or unattended cholera case management. In addition to the possible impact on the cholera outbreak pattern and high case fatality, the COVID-19 pandemic delayed the process of NCP finalization and endorsement. Despite the COVID-19 pandemic challenges, the Ethiopian government tried to implement measures for cholera outbreak investigation and containment, establishing facilities for treatment and conducting reactive OCV mass vaccination campaigns with doses secured from the International Coordinating Group and supported by the government of the Republic of Korea through diplomatic channels.

### Implementation Coordination Structure Decision

During the NCP preparation, 2 major questions underwent long discussions. The first was the issue of NCP implementation—that is, whether it should be a stand-alone program like the Ethiopian Dracunculiasis Eradication Program by establishing a structure from national to lower level, or integrated within the existing health structure. Both approaches have advantages and disadvantages. The stand-alone program approach suggests dedicated staff and offices for implementing and tracking NCP; the integration approach assumes existing health structure with supporting staff for implementation of the NCP. The second discussion was on the establishment of the NHSC. This coordination platform was one of the contentious topics during the plan development. The NCP, in line with the NAPHS, assumed the NHSC coordination platform. However, the NHSC establishment was not succeeded, and the government wanted to use DRMC as a coordinating body during emergencies, under the Deputy Prime Minister Office. The experience showed that nutritional coordination was the major intervention done by this institution.

### Financing the NCP Implementation

A key thrust of the strategy to end cholera by 2030 is to improve WaSH infrastructure and services in cholera hotspots, which requires a sustainable financing mechanism. The 8-year NCP plan forecasted approximately US$404.2 million in need, which includes over US$222.7 million (55% of total NCP budget estimates) for WaSH, US$149.3 million (37%) for case management, US$15.5 million (4%) for OCV vaccination, US$10.8 million (3%) for surveillance, US$3.3 million (1%) for RCCE, and US$2.6 million (1%) for leadership and coordination. In the initial 2 years of the implementation period, a total of US$203.7 million was needed to meet the planned targets. However, a negligible amount was secured that was primarily used for NCP implementation in identified hotspot woredas. Both the available government and international donor contributions were allocated for the cholera outbreak response, covering case management, WaSH, community engagement, and reactive OCV administration. Financial support from the European Union and other partners also extended to address additional humanitarian crises beyond the cholera outbreak response [20]. The 2023 Ethiopia Humanitarian Response Plan calls for US$3.99 billion to assist >20 million people nationwide [21]. The heightened demand for humanitarian crisis response significantly impacted the achievement of planned NCP targets in the first 2 years of NCP rollout, with minimal or no financial resources available. Continuous efforts in national and international stakeholder engagements to gain financial or/and in-kind support are crucial for the success of the Ethiopian NCP goal achievements.

### Access to OCV for Targeted Hotspot Woredas

The Ethiopian NCP laid out a 5-year OCV plan of 2-dose (30 604 889 doses) and single-dose (3 031 266 doses) vaccination in cholera hotspots prioritized [12]. In November 2021, Ethiopia obtained the Global Task Force on Cholera Control’s approval of 6 814 410 OCV doses to conduct preemptive mass OCV vaccination campaigns in 3 phases, targeting 29 hotspot woredas for the first year of NCP implementation [22]. However, these vaccines were not secured due to the global OCV stockpile shortage, largely due to the resurgence of multiple cholera epidemics worldwide and the high demand for OCV doses for reactive vaccinations in outbreak settings. The annual breakdown of OCV dose requirements for the preemptive use in cholera hotspots has been forecasted at around 6 million doses each year [12]. However, these OCV doses planned for preemptive vaccination have been delayed (put on hold), and instead the government secured and administered approximately 7.5 million doses for a series of reactive OCV mass vaccination campaigns for urgent cholera outbreak controls, using a single-dose modality [8].

### Recurrent Cholera Outbreaks and Humanitarian Crises

Ethiopia is one of the most drought-prone countries in the world. The combination of armed conflict, climate shocks, disease outbreaks, and the socioeconomic impacts of COVID-19 have led to the deterioration in humanitarian conditions in the country. As of the end of 2023, approximately 20.1 million people, including an estimated 4.6 million internally displaced individuals, required urgent basic needs support [23]. In addition to this pressing humanitarian need, a nationwide cholera outbreak demands immediate response, particularly in terms of access to clean and safe WaSH services. Both cholera outbreaks and humanitarian responses have been the country's main focus, limiting resources for the much-needed long-term WaSH infrastructure development in cholera hotspot areas [24].

## CONCLUSIONS

Ethiopia prepared the NCP for 2022–2028, intending to reduce cholera fatalities by 90% by 2028, 2 years earlier than the global roadmap target by 2030. The plan is developed through a multisectoral approach involving national and international health and nonhealth ministries and agencies. It includes a targeted approach, identifying 118 cholera hotspot woredas across Ethiopia, with implementation integrated into the existing health structure system at the national and regional levels. The interventions are built upon a comprehensive historical and current situational analysis on cholera outbreak response and preparedness. The plan also harmonizes with other national plans and has a clear high-level coordination platform. The NCP development highlighted the importance of continued investments in quality data collection and health information management systems in cholera control and prevention measures, particularly in the field of cholera surveillance, case management, and use of OCV and vaccine coverage. To achieve the planned NCP targets, the following key points should be addressed going forward: (1) Adequate OCV doses should be ensured for reactive vaccination to control cholera outbreaks and preemptive vaccination in the identified cholera hotspot areas; (2) considering the likelihood of protracted humanitarian crisis in the country, the financial support for NCP rollout should be guaranteed independent of the humanitarian response funds; (3) commitments from the national and international stakeholders for NCP implementation should be sustained and maintained; (4) available financial resources should be used for interventions custom-tailored to each cholera hotspot area or subareas identified for more efficient and effective use of limited funds; (5) a mechanism should be established to ensure the commitment and accountability of each sector in achieving the NCP targets for each pillar of multisectoral approach; and (6) regular review and monitoring of interventions deployed under the NCP should be conducted to assess and track the target goals and activities.

## Notes

***Author Contributions.*** Ethiopia Public Health Institute colleagues, including the authors of this article, have worked on the National Cholera Elimination Plan development, finalization, and rollout. S. E. P. suggested and initiated the write-up of this article. M. K., Y. W. D., and M. E. drafted the initial article. M. K., A. G., Y. J., and S. E. P. revised the article. All authors reviewed and shared feedback on the final article.

***Financial support*.** This study was supported by the Korea Support Committee for International Vaccine Institute (CHMTD05083-010), LG Electronics (CHMTD05083-020), and Community Chest of Korea (CHMTD05083-030). The International Vaccine Institute (IVI) acknowledges its donors, including the government of the Republic of Korea and the Swedish International Development Cooperation Agency.

***Supplement sponsorship.*** This article appears as part of the supplement “Ethiopia Cholera Control and Prevention (ECCP): Evidence-Generation Towards Global Roadmap to Ending Cholera,” sponsored by the IVI.
